# Extrapolation of cytotoxic masked effects in planar in vitro assays

**DOI:** 10.1007/s00216-024-05302-z

**Published:** 2024-04-24

**Authors:** Timothy Rosenberger, Anna Maria Bell, Georg Reifferscheid, Kilian E. C. Smith, Andreas Schäffer, Thomas A. Ternes, Sebastian Buchinger

**Affiliations:** 1 Department G - Qualitative Hydrology, Federal Institute of Hydrology (BfG), Am Mainzer Tor 1, 56068 Koblenz, Germany; 2 Environmental Chemistry - Department of Water, Environment, Construction and Safety, University of Applied Sciences Magdeburg-Stendal, Breitscheidstraße 2, 39114 Magdeburg, Germany; 3 Institute for Environmental Research, RWTH Aachen University, Worringerweg 1, 52074 Aachen, Germany

**Keywords:** Cytotoxic masking, Effect extrapolation, Planar in vitro assays, Chromatogram evaluation, Quantification

## Abstract

The masking of specific effects in in vitro assays by cytotoxicity is a commonly known phenomenon. This may result in a partial or complete loss of effect signals. For common in vitro assays, approaches for identifying and quantifying cytotoxic masking are partly available. However, a quantification of cytotoxicity-affected signals is not possible. As an alternative, planar bioassays that combine high-performance thin layer chromatography with in vitro assays, such as the planar yeast estrogen screen (p-YES), might allow for a quantification of cytotoxically affected signals. Affected signals form a typical ring structure with a supressed or completely lacking centre that results in a double peak chromatogram. This study investigates whether these double peaks can be used for fitting a peak function to extrapolate the theoretical, unaffected signals. The precision of the modelling was evaluated for four individual peak functions, using 42 ideal, undistorted peaks from estrogenic model compounds in the p-YES. Modelled ED_50_-values from bisphenol A (BPA) experiments with cytotoxically disturbed signals were 13 times higher than for the apparent data without compensation for cytotoxicity (320 ± 63 ng versus 24 ± 17 ng). This finding has a high relevance for the modelling of mixture effects according to concentration addition that requires unaffected, complete dose–response relationships. Finally, we applied the approach to results of a p-YES assay on leachate samples of an elastomer material used in water engineering. In summary, the fitting approach enables the quantitative evaluation of cytotoxically affected signals in planar in vitro assays and also has applications for other fields of chemical analysis like distorted chromatography signals.

**Graphical abstract:**

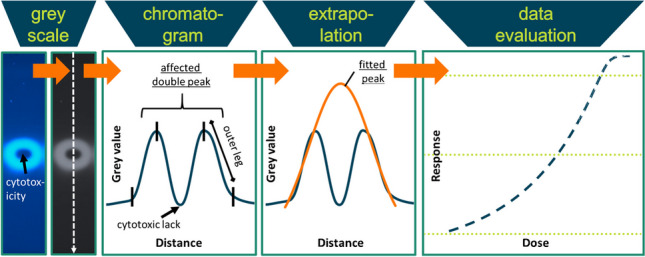

**Supplementary Information:**

The online version contains supplementary material available at 10.1007/s00216-024-05302-z.

## Introduction

Chemical analysis is widely used for environmental monitoring, and its reliability for the identification of pollutants has increased over the past decades. State-of-the-art high-resolution and non-target techniques [1–4] can detect thousands of substances in aquatic (and other) ecosystems at concentrations from the ng/L down to even the pg/L range. Complementary to chemical analysis, in vitro assays provide an appropriate environmental assessment tool, by measuring sum effects of complex samples with multiple analytes [1, 5]. In vitro assays such as the yeast estrogen screen (YES) are well-established and very sensitive tools for the detection of mechanism-specific effects, in this case estrogen receptor activation in different types of samples.

After the standardization of the lyticase-assisted yeast estrogen screen (L-YES) in 2018 (ISO 19040–1) [6], the assay has also become usable for regulatory purposes. However, assays based on the activation of cellular processes, such as the expression of a reporter gene, may suffer from cytotoxicity which masks the detection of a specific effect as a receptor activation due to the presence of dead and thus metabolically inactive cells [7–12]. Frische et al. [10] have addressed this problem for a common mechanism-specific assay and adapted the common YES to investigate cytotoxicity and estrogenicity at the same time. They reported lower estrogenic responses due to masking effects caused by cytotoxicity. They also pointed out that the importance of cytotoxicity “should not be considered as mutually exclusive approaches for the estrogenicity assessment”.

In the recent past, planar bioassays combining high-performance thin layer chromatography (HPTLC) for a fractionating of the sample components with cell-based methods, e.g. the planar yeast estrogen screen (p-YES) [9, 13–15], were developed. This approach can be used in different contexts including food safety [16], environmental issues [8] and for effect-directed analysis [9, 17]. A broad spectrum of endpoints have been already established for such planar in vitro assays*.* Endpoints include estrogenicity, androgenicity, anti-androgenicity, thyroidogenicity, dioxin-like effects, effects on the vitamin D receptor, effects on the retinoic acid receptor, genotoxicity, luminescent bacteria toxicity, photosystem II and acetylcholinesterase inhibition [7, 8, 13, 14, 17–20].

Planar in vitro assays circumvent the above-mentioned cytotoxic masking to a certain extent, by detecting effects along a mass gradient of the signal spots, i.e. the spatial distribution of the analyte on the HPTLC-plate. A number of studies have reported the parallel detection of cytotoxicity and mechanism-specific toxicity in planar in vitro assays [7–9, 16]. Cytotoxicity caused by compounds at higher amounts results in typical ring pattern signals, also called “haloes” [7, 16], due to a diffusion dependent analyte mass gradient from the inner to the outer area of the analyte spot [9, 16]. The inner area of the ring signal contains the highest amount of a potentially cytotoxic analyte, which leads to a lacking signal response. The visible signal of the outer ring contains decreasing amounts of the analyte at a non- or less cytotoxic level. Shakibai et al. [7] mention the advantages of planar assays, which are able to detect specific effects in the presence of cytotoxic chemicals, over multi-well plate assays. Furthermore, Riegraf et al. [20] developed an advanced planar assay to distinguish between cytotoxicity and anti-androgenicity but did not focus on how to deal with actual cytotoxically affected signals in planar assays. This indicates the lack of a method to quantify signals in planar assays when cytotoxicity occurs.

Signals detected by planar in vitro assays are converted to chromatograms based on measured pixel intensities along a sample track. Thus, ring-shape signals resulting from cytotoxic effects lead to double peaks. A simple quantification of these signals as a sum of pixel intensities is not meaningful, due to the lack of signal information in the centre of the ring-signal with the highest amount of the target analytes. The calculation of a biological equivalence concentration based on an affected peak would lead to an underestimation of the effect. In consequence, it is not possible to generate complete dose–response relationships for compounds exhibiting both specific and cytotoxic effects. Due to the lack of a complete dose–response relationship, a meaningful calculation of effect-dose values (ED_x_) is hampered by an undefined maximal effect level that would be used to normalize effects to 100%. Thus, the calculation of relative potencies and/or the toxicity of an analyte or chemical mixture might be not possible or impacted by higher uncertainties [21–23]. The aim of this study was therefore to develop a method for the extrapolation of cytotoxically affected signals in planar in vitro assays and to generate sufficient data to quantify the effect signals. The underlying assumption is that the outer legs of the double peak contain sufficient information to model a peak that reflects the unaffected signal, i.e. the signal that would result without overlaying cytotoxicity by using a suitable peak function such as the Gaussian, exponentially modified Gaussian, Lorentzian or log-normal functions.

## Experimental

### Chemicals

Bisphenol A (BPA, CAS: 80–05-7, ≥ 99%), estrone (E1, CAS: 53–16-7, ≥ 99%), 17β-estradiol (E2, CAS: 50–28-2, ≥ 98%), 17α-ethinylestradiol (EE2, CAS: 57–63-6, ≥ 98%), estriol (E3, CAS: 50–27-1, ≥ 97%) and 4-methylumbelliferyl-β-D-galactopyranoside (MUG, CAS: 6160–78-7) were obtained from Sigma-Aldrich. N-hexane (CAS: 110–54-3, ≥ 98%) was obtained from Supelco; methanol (CAS: 67–56-1, ≥ 99.9%) from Merck; ethanol (CAS: 64–17-5, ≥ 99.8%) from Riedel-de-Haen; ethyl acetate (CAS: 141–78-6, ≥ 99.5%), n-heptane (CAS: 142–82-5, Picograde) and acetone (CAS: 67–64-1, Picograde) from Promochem; and chloroform (CAS: 67–66-3, 99.0–99.4%) from Honeywell.

### Standard solutions

BPA solutions were prepared from 1.9 µg/ml to 1 mg/ml in ten 1:2 dilution steps. Estrogen mixtures were prepared from 0.2 to 40 ng/ml for E1, from 0.02 to 4 ng/ml for EE2 and E2 and from 2 to 400 ng/ml for E3 (“Supplementary information”, Table S1). Dilutions were made with ethanol.

### Extract of a building material leachate

To further apply the present approach in a real scenario, a sample from an elastomer used *inter alia* in water engineering was selected. This sample was chosen due to the occurrence of cytotoxically disturbed signals in the p-YES. The test sample was produced by an aqueous leaching test, which was part of an investigation of building materials for their environmental sustainability. Three replicate slices of the elastomer were fixed on nylon strings, placed in glass tanks (300 × 220 × 240 mm) and submerged in deionized water with a volume to surface ratio of 33 L/m^2^. The tanks were covered with glass lids. Three additional blank replicates containing only deionized water were also prepared. Leachates were sampled after 4 weeks and stored in dark glass bottles at 2–8 °C. OASIS HLB 6cc (200 mg) solid-phase extraction cartridges were conditioned with 2 ml n-heptane, 2 ml acetone, 3 × 2 ml methanol and 4 × 2 ml double distilled water. The cartridges were then used to concentrate the leachates 500-fold by loading them with 1000 ml leachate. After a drying step the cartridges were eluted with 4 × 2 ml methanol. The extracts were reduced by evaporation and finally made up to 2 ml with methanol. For testing, the samples were diluted with methanol to the concentrations mentioned.

### HPTLC

MERCK silica gel 60 F_254_ (20 × 10 cm) plates were used to perform normal phase HPTLC for the samples and the estrogen standard mix. Plates were pre-treated according to Schoenborn et al. [13]. The plates were prewashed with 5 ml methanol to 5 mm below the upper rim before usage. Subsequently, the plates were activated at 110 °C for 30 min and stored in aluminium foil in a desiccator until use.

Application of leachate samples and standard solutions was performed with a CAMAG TLC Sampler ATS 4. Analytes were applied at 8.0 mm Y-position with a band length of 5.0 mm. For standard solutions, volumes of 5 µl for the elastomer sample, 1 µl of the 0.5 × concentrate and 1; 2; 5; 7.5; 10; 15 and 20 µl of the 1 × concentrates were applied. Samples were focused according to Spira et al. [15] with methanol up to 20 mm, and dried for 3 min. Subsequently, the plates were developed according to a slightly modified procedure from Cimpoiu et al. [24] with 20% ethyl acetate, 25% n-hexane and 55% chloroform to 90.0 mm in a CAMAG AMD 2. For documentation of the intermediate steps, images of the plates were made with a CAMAG Visualizer 2 at white light, 254 nm and 366 nm. BPA standard amounts were applied at 50.0 mm (Y-position) on separate plates and the plates were not chromatographically developed.

### Yeast cultures

The *Saccharomyces cerevisiae* strain according McDonnell et al. [25, 26] was cultivated for testing. Overnight culture and exposure media were prepared according to ISO 19040–1 [6]. Overnight cultures were inoculated and incubated over 20–22 h at 30 °C in an orbital shaker (IKA^®^KS 3000 i control) at 200 rpm. Subsequently, test cultures were prepared by pelleting (centrifugation at 2500 g for 10 min) and resuspending in test medium. The test cultures were finally adjusted to a FAU (Formazine Attenuation Units) of 1500 ± 100 at 600 nm according to ISO 7027 [27] with a Tecan Infinite 200 PRO.

### Planar yeast estrogen screen

A detailed procedure of the p-YES is described in Schoenborn et al. and Riegraf et al. [8, 28]. Once the HPTLC plates were developed, the yeast the suspension and MUG mixture was applied with a CAMAG Derivatizer. HPTLC plates were treated with 3 ml of yeast suspension (yellow nozzle, spray intensity 5) and incubated at 30 °C and 90% relative humidity for 3 h in plastic boxes (NuAire CO2-incubator, NU-5820E). Afterwards, 2.5 ml of the MUG mixture solution was applied (green nozzle, spray intensity 5), and the plates were incubated for a further 15 min at 37 °C (Memmert IPP 400) without a lid. Finally, images were taken at 366 nm in high-quality mode with a CAMAG Visualizer 2. P-YES testing was conducted with the BPA standard, the estrogen mixture and the leachate sample for a minimum of three replicates. To confirm cytotoxicity in the BPA experiments, an additional resazurin assay was performed after Riefrag et al. [20]. For detection of cytotoxicity, an elongation of the exposure time from 3 to 20 h was necessary.

### Python modules used

To achieve an extrapolation of cytotoxically affected estrogenic signals, a program was developed in Python (version 3.9.12) [29] using the Integrated Development Environment “Spyder 5” (Version 5.1.5) [30]. The resulting code is available on GitHub under the following Link:

https://github.com/bafg-bund/extrapolation-cytotoxic-masking

In addition, Python was used for univariate statistics and generating box- and scatterplots. The package “Pillow” (Version 9.0.1) [31] and its module “ImageOps” were used for image processing and grey value calculation. The package “NumPy” (Version 1.21.5) [32] was used for numerical processing, “SciPy” (Version 1.7.3) [33] and its modules “special”, “signal”, “stats” and “integrate” were used for the error function, signal finding, peak integration and performing of univariate statistics. For post hoc testing, we made use of the “scikit_posthocs” (Version 0.7.0) [34] package. “Pandas” (Version 1.4.2) [35, 36] was used in the case of data frames (tabular arrays). Peak modelling and parameter estimation were performed with the module “SciPy.optimize”. For data plotting, we used the module “matplotlib.pyplot” (Version 3.5.1) [37]. “Shapely.geometry” (Version 1.8.5) [38] was used for its “LineString” function to vectorize and find intersections of data points and the package “openpyxl” (Version 3.0.9) [39] for creating and manipulating Excel workbooks and -sheets. The built-in python modules “random” and “datetime” were used for the random function and reading the operating system time [29].

### Image processing

P-YES images received from the Visualizer were imported and processed to grey images (8-bit scale). Grey values along a sample track were then averaged over 50% of the originally sample application width. The average grey values were processed to chromatograms and further processed with a Savitzky-Golay filter as mentioned by Ristivojević et al. [40]. Filter conditions were set to “polyorder” = 2 and “window_length” = 111. These conditions were found to provide data with low noise and without over-smoothing. For this reason, they were used for all chromatograms. The resulting chromatograms were employed for peak integration and effect modelling.

### Peak integration

To eliminate background noise, the program calculates integrals of user-defined linear base lines in the range of an user-defined peak area of choice. Subsequently, base integrals are subtracted from raw peak integrals in order to achieve the actual peak integrals.

The data base for the modelling was also obtained from a user-defined peak area. As for the integration of undisturbed peaks, a base integral was also subtracted from modelled peaks to ensure adequate peak integrals. In addition, when the course of the modelled peak built intersections with the baseline, the base integral was calculated within the intersections in order to avoid overestimation of the base integral.

### Peak functions

Four peak functions were implemented for effect modelling (Eq. 1 to Eq. 4). In Eq. 1 to Eq. 4, *A* is the peak amplitude, *x* is the specific track position, *xr* is the peak width, *µ* is the mean peak position, *σ*^*2*^ is the variance, *σ* the standard deviation for an elevated number of data points, and *α* is the respective peak shape parameter. Peak functions were then validated as described in Fig. 2 and “Results of the approach validation” section.

Gaussian function (Gaussian) [41].

1 $$y\left(x\right)=A{\text{exp}}\left[\frac{-{\left(x-\mu \right)}^{2}}{{\sigma }^{2}}\right]$$

Exponentially modified Gaussian function (modified Gaussian) [42, 43].

2 $$y\left(x\right)=\frac{\sqrt{2\uppi }A\sigma }{2\sigma }{\text{exp}}\left[\left(\frac{{\sigma }^{2}}{2{\alpha }^{2}}\right)+\left(\frac{xr-x}{\alpha }\right)\right]\left[1+{\text{erf}}\left(\left(\frac{x-xr}{\sqrt{2}\sigma }\right)-\left(\frac{\sigma }{\sqrt{2}\alpha }\right)\right)\right]$$

Lorentzian function (Lorentzian) [44].

3 $$y\left(x\right)=\frac{2A}{\uppi }\left(\frac{y}{4{\left(x-\alpha \right)}^{2}+{y}^{2}}\right)$$

Log–normal function (log-normal) [42, 45].

4 $$y\left(x\right)=A{\text{exp}}\left[\frac{-{\text{ln}}\left(2\right)}{ln{\left(\frac{\alpha }{\sigma }\right)}^{2}}{\text{ln}}{\left(\frac{x-xr}{\sigma +\alpha }*\frac{\frac{\alpha }{{\sigma }^{2}}-1}{\frac{\alpha }{\sigma }}+1\right)}^{2}\right]$$

### Data to validate the approach

The suggested approach was validated by fitting these four peak functions to 42 ideal peaks. The unaffected signals were obtained from the p-YES by applying estrogen standard mixtures in amounts of 1–10 pg E1, 0.1–1 pg EE2, 0.1–1 pg E2, 10—100 pg E3 and 10 and 20 ng BPA standard solution (see “Standard solutions” section) as agonists of the estrogen receptor. These did not lead to affected signals in the applied ranges. The ideal peaks were further used to generate mathematically generated affected double peaks (see Fig. 2). The double peaks were generated by subtracting Gaussian data from ${~}^{2}\!\left/ \!{~}_{3}\right.$ of the center of the entire original peak. The parameters were adjusted according to Eq. 1 as follows: the peak amplitude (*A*) was set to half the maximum value, the mean peak position (*µ*) was determined as the corresponding *y*-value of the midpoint *x*-value, and the variance estimated by the standard deviation (*σ*) was set to ${~}^{1}\!\left/ \!{~}_{5}\right.$ of the length of the data set to obtain Gaussian curves which are thinner than the ideal peaks.

### Dose–response estimation, statistical testing

The measured and calculated integrals were further processed with “R” (version 4.2.2) and the package “drc” (version 3.0–1) [46, 47] to calculate dose–response parameters. For dose–response curves, the data were normalized to the maximum value within all replicates, and means were calculated for each dose signal [17]. The mean data were then fitted with a five parametric function (Eq. 5, *x* is the concentration, *d* is the bottom plateau, *a* the top plateau, *w* is the inflection point, *b* is the slope, and *f* is the symmetry factor). The integral data were tested for normal distribution using a Shapiro–Wilk test. In addition, a Levene’s test was performed for evaluation of the homogeneity of variance. Differences in integral data were examined by performing a Kruskal–Wallis and posteriori a Dunn’s test.

Used five parametric function for the dose–response calculations [46–48].

5 $$f\left(x\right)=d+\frac{a-d}{{\left(1+{\left(\frac{x}{w}\right)}^{b}\right)}^{f}}$$

For the validation, outliers were identified using a Hampel’s test. In addition, chi-square tests were performed for these results. The chi-squared analysis serves as a measure for the goodness of the fit by comparing measured and fit data. A *p*-value closest to 0.5 represents the best fit, and values between 0.05 and 0.95 were considered to be sufficient.

## Results

### Rationale for the approach

To create a data basis, bisphenol A was chosen as an analyte which showed both estrogenic and cytotoxic effects especially at amounts above 640 ng.

**Fig. 1:**
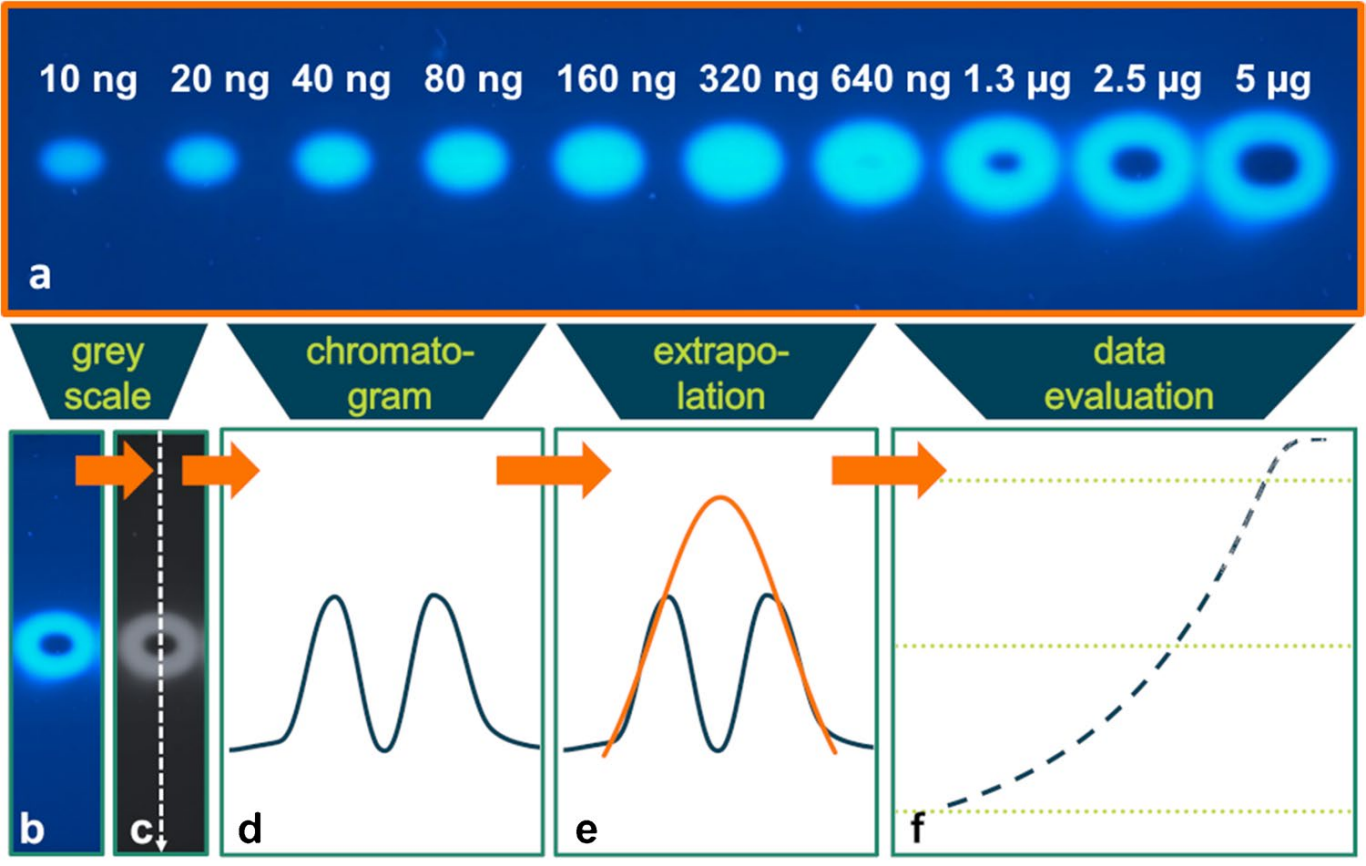
**a** Image of 10 p-YES effect signals of BPA spotted on a TLC plate (without chromatographic development). Amounts ranged from 10 ng to 5 µg. At 1.3 µg and above, cytotoxic effects on the signals are clearly visible. The image shows the signal detection with fluorescence imaging at *λ*_excitation_ = 366 nm. **b**–**f** Diagram illustrating the proposed approach for the extrapolation of cytotoxically affected peaks. **b** Image of cytotoxically affected p-YES effect signals resulting in “ring” patterns. **c** Transformation of the signals to grey scale images. **d** Generation of chromatograms along the sample tracks and plotting of grey values against track distance. As can be seen, double peaks result from cytotoxically affected signals. **e** Fitting a peak function to the outer legs of the peaks. **f** Evaluation of the dose–response relationship using a dilution series of the sample. **d** and **e** *x*-axis = distance, *y*-axis = grey value. **f** *x*-axis = dose, *y*-axis = response

Figure 1a shows, by way of example, signals with cytotoxic affection generated by the analysis of BPA in the range from 10 ng to 5 µg. Cytotoxicity affected signals are clearly visible at an applied amount of 1.3 µg BPA and above. By creating chromatograms from these images, double peaks are acquired for affected signals, which do not allow for the adequate quantification of the effect signals. The following approach was used to develop a method for the extrapolation of cytotoxic masked effects by the modelling of unaffected signals using a peak function.

Four functions (Gaussian, exponentially modified Gaussian, Lorentzian and log-normal; Eq. 1 to Eq. 4), generally used for chromatogram evaluation (see “Peak functions” section), were selected to model ideal signals. Due to their suitability for also asymmetric shapes (including fronting and tailing) [42, 49, 50], the modified Gaussian and the log-normal functions are often used for chromatographic peak modelling in both gas and liquid chromatography [51–54]. In contrast, the Gaussian and Lorentzian functions are more commonly used for symmetrical modelling [42], and both are peak functions used in liquid and gas chromatography [55–59]. The Lorentzian function is also frequently used for Raman spectrometry [60, 61].

**Fig. 2:**
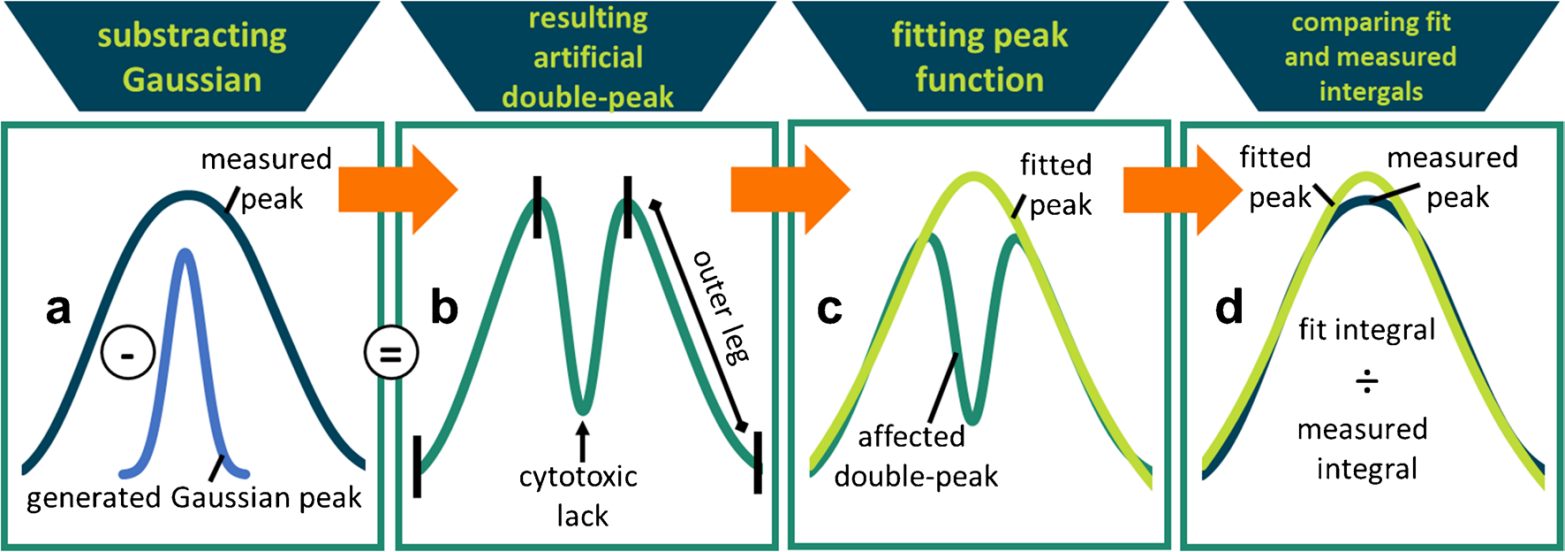
Scheme showing the mathematical generation of simulated cytotoxically affected p-YES effect peaks based on ideal signals and the calculation of integral ratios. **a** Subtracting of individually generated Gaussian peaks from measured peaks. **b** Resulting double peak, bars indicate the outer leg areas. **c** Fitting of the respective peak function to the generated double peak. **d** Calculation of ratios from fit integrals to measured integrals

The aim of the study was to develop an algorithm that uses the remaining information of cytotoxically affected signals and to extrapolate the actual specific response. Chromatograms were generated as described in “Image processing” and Fig. 1. The approach works by using the remaining data of the affected peak signals. The outer legs of the resulting double peaks caused by cytotoxicity are used as the data basis to fit the undisturbed peak signal and to extrapolate masked effects. The outer peak legs defined in Fig. 2b are automatically detected by the algorithm by finding the two maxima of the double peak. If the extrema could not be detected automatically, the outer legs were defined manually. This occurred once for the elastomer sample signals. The data from the outer legs were used for curve fitting with the peak function of choice and the least squares regression. Estimated parameters of the fitted function were used to extrapolate the impacted part of the affected signal.

Firstly, the approach was validated by using the 42 ideal signal peaks (see “Data to validate the approach” section). The peaks showed no plateaus due to the technically limited 8-bit camera or on-plate analyte overload in the chosen dose ranges. The modelled and measured peak integrals (the original unmodelled peak integrals) were compared as further detailed in “Results of the approach validation”. In a second step, the measured ideal signals were mathematically transformed to simulate cytotoxically affected peaks by subtracting individually generated Gaussian peaks from the measured data (see “Data to validate the approach” section and Fig. 2a and b). In this way, it was possible to validate the modelling of affected peaks using the outer legs of the signal, by comparing the integrals of fitted double peaks to the original integral.

The developed method then was applied to a dose–response relationship of BPA as a model compound that activates the estrogen receptor but is also cytotoxic at doses above 1.3 µg. The resulting dose–response relationships using (a) the measured raw, i.e. affected, signal and (b) the modelled signal were compared in terms of calculated EDx-values for BPA. Finally, the method was used to evaluate the potential toxicity of an elastomer using the p-YES assay.

### Results of the approach validation

#### Step 1: ideal peak fitting

**Fig. 3:**
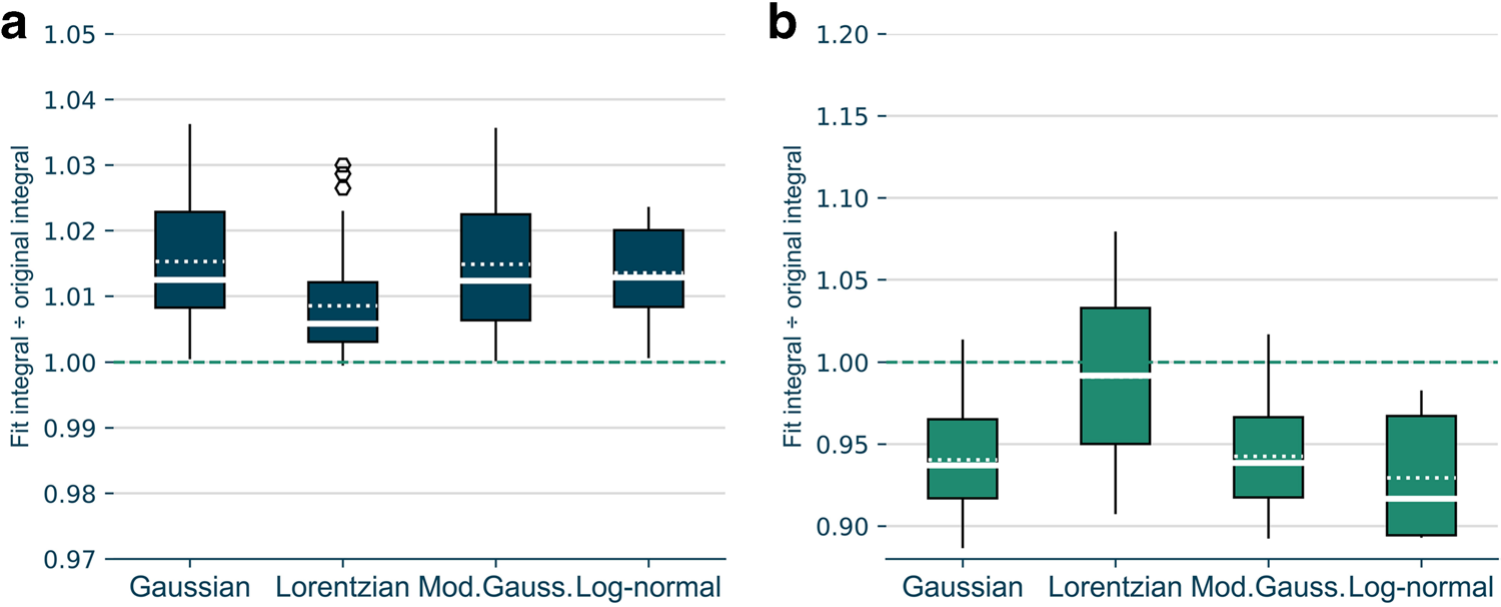
Ratios of **a** p-YES effect peak integrals of reference compound (BPA, E1, E2, EE2 and E3) spots on a HPTLC plate to measured integrals of ideal signals and **b** fit integrals from mathematically constructed, affected peaks to measured integrals of ideal signals. The closer the ratio is to the value of 1.0, the better the alignment of the fitted peak function integral to the measured integral. Boxes show the first and third quartiles, dashes show medians, dotted dashes show means and hexagons show values outside the 1.5-fold interquartile range indicated by Whiskers. The optimal ratio of fit-integrals to ideal signal integrals of 1.0 is marked with a green dashed line. *n* = 42 for Gaussian, Lorentzian and mod Gaussian, *n* = 8 for log-normal

In the first validation step the aim was to find the most appropriate peak function (see “Peak functions” section) to best reproduce the original p-YES peaks. Therefore, the ratios of the measured peak integrals to the integrals resulting from the peak function fits were calculated (illustrated in Fig. 2d). The closer the ratio is to the value of 1.0, the better the alignment between the modelled and measured peak area. Figure 3a shows the data of all 42 ratios (see “Supplementary information”, Table S4 for corresponding values) for modelling with the four functions as boxplots.

The Lorentzian mean ratio differed least from 1.0 (0.9%). This was followed by the log-normal (1.4%), Gaussian and modified Gaussian (1.5% each). The data showed no normal distribution. Therefore, a Kruskal–Wallis test (*p*-value = 0.001) and a subsequent Dunn’s post hoc test were performed for statistical analysis. The results show that the Lorentzian function significantly differed from the Gaussian and modified Gaussian ratios (*p*-value =  < 0.001 and 0.001). The 1.5-fold interquartile ranges differed by a maximum of 2% for all functions used. The values outside the 1.5-fold interquartile range in Fig. 3a were not identified as outliers by the Hampel’s test. For the Gaussian, modified Gaussian and log-normal extrapolations, all calculated ratios were above the value of 1.0. In the case of the log–normal function, the fitting was successful for only 8 of the total 42 peaks (19%).

In addition, chi-squared tests were performed to assess the goodness of the fits (see details in “Supplementary information”, Fig. S2). The Gaussian function showed a mean *p*-value for the chi-squared test closest to 0.5 (0.73) followed by the log–normal function (0.86), the Lorentzian (0.92) and modified Gaussian functions (0.94). The values marked with hexagons (outside 1.5-fold interquartile range) in Fig. S2 (“Supplementary information”) were not identified as outliers by the Hampel’s test for the Gaussian function. For the Lorentzian and the modified Gaussian functions, one outlier each was identified by the Hampel’s test.

#### Step 2: mathematically simulated cytotoxicity

In the second validation step, the extrapolated signals were validated for the performance of the respective peak function for the extrapolation of cytotoxicity affected signals. In analogy to the previous step, the integrals of the fits resulting from the simulated cytotoxicity peaks were related to the integrals of the original unaffected peaks by calculating quotients of the respective integrals (Fig. 3b). Again, the closer the ratio approaches 1.0, the better the alignment between the modelled and measured peaks. For the Gaussian, Lorentzian and modified Gaussian functions, boxplots based on all 42 ratios are shown. In case of the log-normal fits, only 8 ratios were used for the boxplot because the fitting was not successful for the remaining 34 signals (see “Supplementary information”, Table S4).

The ratios obtained using the Lorentzian function showed a mean deviation of 0.9% from the optimal value of 1.0. This is the lowest deviation obtained for the four functions tested, followed by the mod Gaussian (deviation 5.7%), the Gaussian (deviation 5.9%) and the log-normal ratios (deviation 7.0%). Due to the lack of a normal distribution of the calculated ratios, a Kruskal–Wallis test was performed (*p*-value =  < 0.001), which again indicated significant differences between the four peak functions. The Dunn’s test indicated that the Lorentzian ratios significantly differed from all of the other functions (*p*-values: Gaussian =  < 0.001, mod Gaussian =  < 0.001, log-normal =  < 0.001). More than 90% of all mean ratios for the Gaussian, modified Gaussian and log-normal showed an underestimation of the actual integral (indicated by ratios below 1.0). In the case of the Lorentzian function, 59% of the modelled signal resulted in an underestimation of the peak integral.

#### Sensitivity analysis

To determine if the proportion of the outer legs used for the modelling (see Fig. 2b) influences the quality of the modelled peaks a sensitivity analysis was performed. Therefore, the percentage of the outer legs used for the modelling was varied in ten steps from 10 to 100% coverage of the outer leg. For this analysis, the data from the “Step 2: mathematically simulated cytotoxicity” section were used. The aim was to verify whether cutting off unnecessary parts of the outer legs (as the flattening transition to local extrema) could improve the quality of the modelling, as it then would be based only on the width of the double peaks and the steepness of the outer legs. The quality is assessed by the ratio of the modelled and measured peak integrals as used in the “Results of the approach validation” section. For this purpose, an algorithm was developed that identified the inflection points of the outer legs as the steepest point of the slope in the outer legs. The ranges (10–90%) of the outer legs used as the data basis for the modelling started from the inflection points. The maximum range (100%) of the outer legs was defined as the whole outer legs detected by the algorithm described in the “Rationale for the approach” section. As detailed in the discussion (see “Validation” section), the Lorentzian function was chosen for all further peak extrapolation. For this reason, we focused the described evaluations only on the Lorentzian function.

A reduction of the data basis resulted in a loss of peak fitting quality. The ratio as a quality measure (preferably a value closest to 1.0) was stable with values just below 1.0 between 100 and 70% data coverage of the outer legs (also see “Supplementary information”, Fig. S3 and Table S3). The modelled peak integral was increasingly overestimated below 60% data coverage. The interquartile range of the calculated ratios increased steadily below a value of 80% data coverage indicating a higher variability with reduced coverage of the outer legs. For practical reasons, the full data range (100%) was selected for all further calculations, as the ratio only differed 0.9% from the optimal value of 1.0. For additional information, see the supplementary data (“Supplementary information”, Fig. S3 and Table S3).

### Impact of affected signals on toxicity measures

#### Cytotoxicity affected signals from bisphenol A

After validating the most appropriate peak function for extrapolation of cytotoxicity affected peak signals, the approach was tested on laboratory generated data that were affected by cytotoxicity. As BPA is known to be both estrogenic [8, 16, 62–64] and cytotoxic [9, 16] to the cultivated yeast strain, BPA was used as a model compound to investigate the impact of affected peaks on toxicity measures such as ED_x_-values. BPA was applied in a range from 10 ng to 5 µg in 10 dilution steps and subsequently analysed by means of the p-YES assay (Fig. 1). The cytotoxicity of BPA was verified for these experiments by a resazurin assay according to Riegraf et al. [20] (see “Supplementary information”, Fig. S1).

**Fig. 4:**
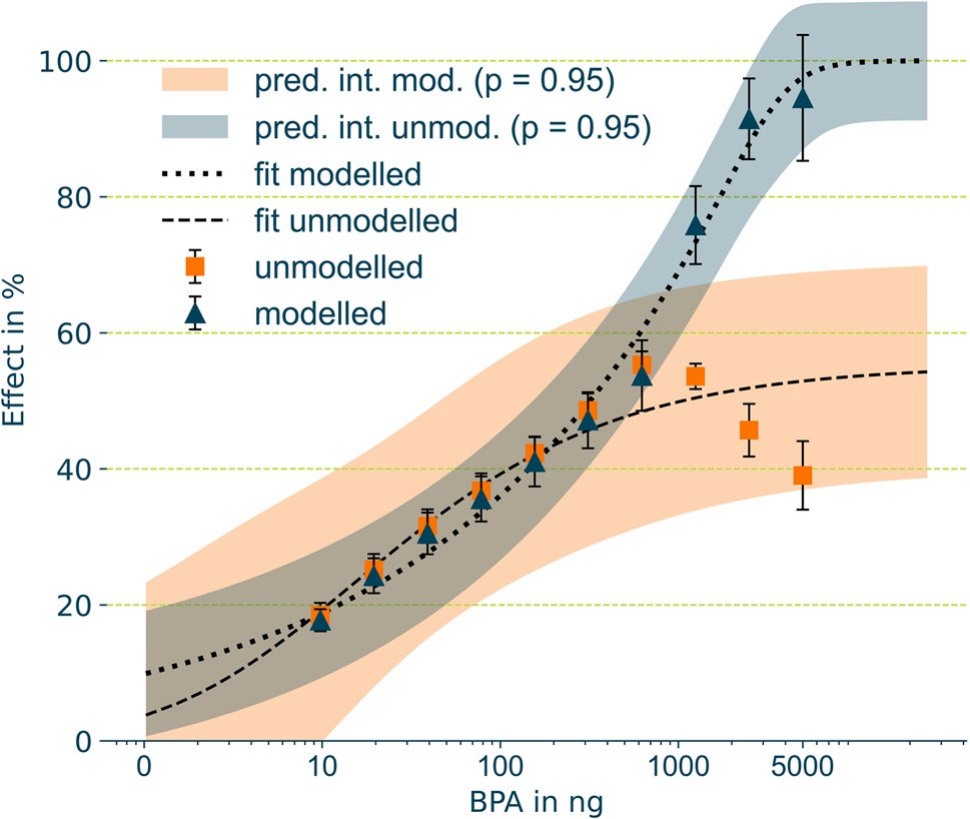
Dose–response relationships of effects in comparison, derived from a five-parametric estimation (Eq. 5) from modelled (mod.) and unmodelled (unmod.) integrals of BPA p-YES signals (*n* = 4). Effects are plotted against logarithmic concentrations. Dashed lines show estimated relationships, markers show the underlying data, error bars show 95% confidence intervals, and bands show 95% prediction intervals (pred. int.)

The resulting dose–response relationships for the unmodelled, i.e. observed, and modelled BPA signals, are shown are compared in Fig. 4. The first seven doses (10 to 640 ng) show almost equal signal intensities when comparing modelled and unmodelled integrals. Differences range from 0.6% at 10 ng up to 3% for 0.3 µg BPA. Increasingly larger differences occurred in parallel with the onset of cytotoxicity, as is evident by the decreased unmodelled signals above 640 ng BPA, which further decreased with increasing amounts of BPA with 51% at 1.3 µg, 111% at 2.5 µg and even 150% at the maximum amount of 5 µg BPA. The ED_50_ value and respective 95% confidence interval for modelled BPA data was 320 ± 63 ng. For comparison, a value of 24 ± 17 ng was determined if the unmodelled signal was used as data for the dose–response relationship. Regarding the endocrine activity of BPA, the extrapolated signal data reveal a lower toxicity than that derived from the uncorrected YES-assay signals.

#### Extract of the eluted elastomer

**Fig. 5:**
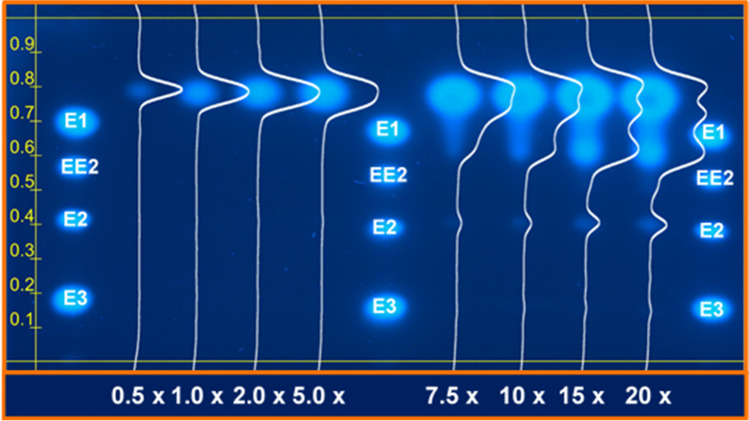
Image of signals from an extract of the eluted elastomer using the p-YES. Samples were applied in different volumes on HPTLC-plate as indicated at the bottom of the figure and subsequently separated using two-step chromatographic development with (1) methanol and (2) ethyl acetate, n hexane and chloroform (20;25;55, V:V:V). 50 pg estrone (E1), 5 pg ethinylestradiol (EE2), 5 pg estradiol (E2) and 500 pg estriol (E3) served as positive controls applied on the right, the middle and the left tracks. The image shows the signal detection with fluorescence imaging at *λ*_excitation_ = 366 nm. In addition, responsive chromatograms derived from plotted grey values are shown superimposed on the signals, and an Rf-scale is displayed on the left edge

To illustrate the application of the proposed approach to a real sample, p-YES results from the elastomer leachate sample were used (see “Extract of a building material leachate” section). Eight increasing amounts of the sample extract were applied from 0.5 to 20 × relative concentration factors (RCF). From 7.5 to 20 × RCF, the typical ring pattern, indicating cytotoxicity, occurred (see Fig. 5). The affected signals showed a mean retention factor (Rf) value of 0.77 ± 0.01. Above an RCF of 7.5 × , further to the cytotoxically affected signal at Rf 0.77, additional signals representing metabolites or further contaminants appeared resulting in three possible signals (signal 2 Rf = 0.60 ± 0.01; signal 3 Rf = 0.39 ± 0.01).

**Fig. 6:**
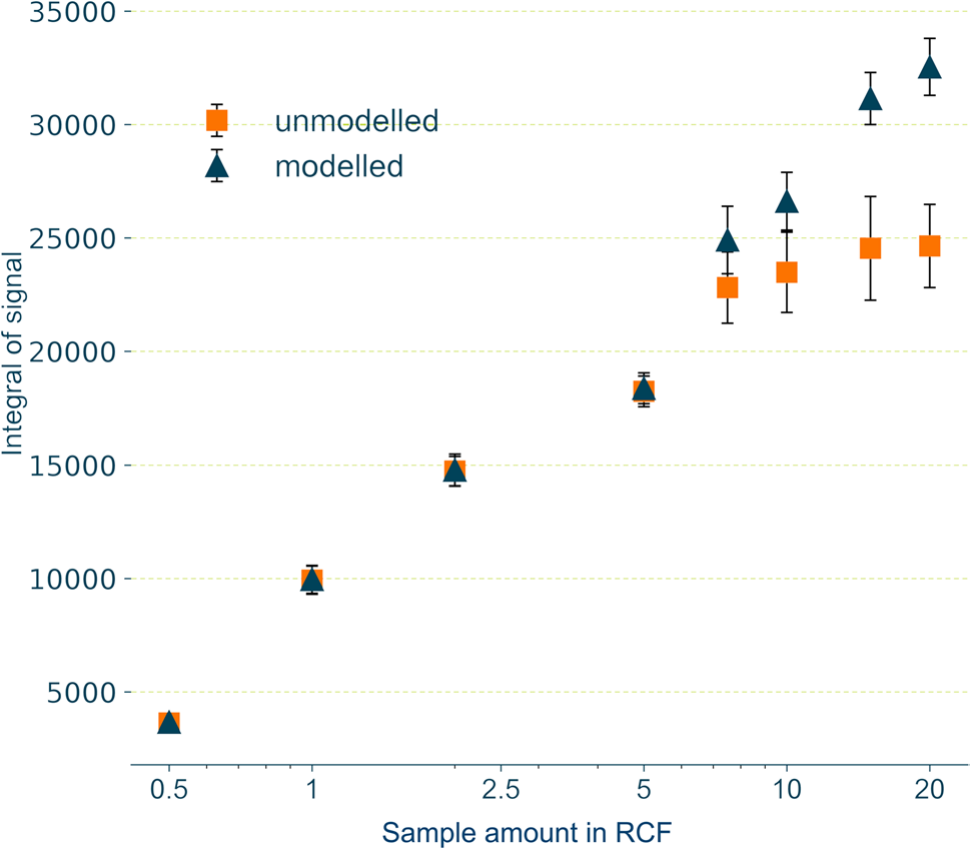
Comparison of effects from modelled and unmodelled integrals of extracted building material leachate (*n* = 3) derived from the p-YES test. Effects are plotted against logarithmic concentrations. Markers indicate data and error bars indicate 95% confidence intervals

Figure 6 shows the resulting integrals with and without correction. In the range from 0.5 to 7.5 × RCF, the integrals for the modelled and unmodelled data showed similar values. Maximal differences of 1% were observed for an RCF of 0.5 × and the lowest difference of 0.3% for an RCF of 1 × . However, for RCF of 7.5 × and above, the differences between modelled and unmodelled data increased (7.5 × : 9%; 10 × : 13%; 15 × : 27%; 20 × : 32%).

## Discussion

The present approach was designed to serve as a tool to compensate for cytotoxically masked signals in planar in vitro assays. Planar assays only circumvent cytotoxicity to a certain extent, and it is not possible to directly use the integrals of the disturbed ring-signals to quantify toxicity.

### Validation

The underlying 42 optimal peaks resulting from spotting BPA and estrogen mixture standards on the HPTLC plates were analysed on different days, but the corresponding peak integrals did not differ by more than on average 3% (maximum 9%) indicating a robust performance of the applied method. Based on this finding, a maximum difference of the fitted integral to the measured integral of 3% was set as threshold for acceptable results in the first validation step. For the second step, a maximal deviation of 9% was accepted.

The ratios of the measured and fitted peak integrals did not exceed the limits of 3% and 9% for any of the functions applied to correct for cytotoxicity. In both validation steps, the Lorentzian function showed a mean ratio closest to 1.0 (both steps 0.9%; see Fig. 3) and were thus far away from the set limits. The log–normal function showed acceptable results for both, ideal and affected peaks, but a determination of meaningful parameters for the function by least squares optimization was only possible for 19% of the data. This might be avoided by adjustments of the presets for the fitting. As the log–normal function performed worst, even in those cases for which a fitting was possible, it was not included in the further evaluation.

Although in the first validation step, the chi-squared analysis indicated a tendency for an over fitting of the measured signal by the Lorentzian function compared to the Gaussian and the log-normal functions (see “Step 1: ideal peak fitting” section and “Supplementary information”, Fig. S2), all functions provided acceptable fittings within a range of *p*-values between 0.05 and 0.95. In the second validation step, in case of the Lorentzian function, 59% of the modelled signals resulted in an underestimation of the peak integral. The reason for this underestimation tendency is probably the lack of information (missing data) from affected signals. Although the Lorentzian function has a wider interquartile range, it still captures the measured integrals better due to its significantly smaller deviation from 1.0.

In sum, it was concluded that the fitting of the signals using the Lorentzian function resulted in the most accurate representation of the data obtained by the bioassay. Thus, the Lorentzian fitting was selected for all further calculations. The validation showed that the approach gives good results for the extrapolation of cytotoxic masked effects.

### Application of the approach

The BPA experiments were performed to generate cytotoxically affected signals and illustrate how cytotoxicity is manifested in ring patterns in planar in vitro assays. Up to 640 ng BPA spotted on HPTLC plate, similar effect results for the modelled and unmodelled data were obtained. Above the amount of 640 ng BPA increasing differences occurred (see Fig. 4). Using the modelled data for the fitting of the dose–response relationship resulted in a sigmoidal dose–response relationship, as would be expected for unaffected signals. The identification of cytotoxicity at higher BPA concentrations with a subsequent resazurin assay (see “Supplementary information” Fig. S1) indicates that the ring patterns observed at higher concentrations were not caused by assay specific interferences such as fluorescence quenching [65] or an inhibition of the reporter enzyme but rather by cytotoxic effects of BPA. However, with respect to the evaluation of toxic properties of a compound, the mechanistic cause of the signal interference is not relevant.

The ED_50_ derived from modelled signals was 13-times higher than that from the unmodelled signals (320 ± 63 ng versus 24 ± 17 ng). Another study [16] reported an ED_50_ value of 4.6 ng under similar conditions (yeast strain and HPTLC plates without development). In addition to variations in the assay performance, one possible explanation for the differences in ED_50_ values might be the use of non-extrapolated signals and a limitation of the tested doses to 570 ng in the previous study [16], a value below the onset of cytotoxic effects above 640 ng BPA in our experiment. The ED_50_ value for BPA determined in the current study is in a similar mass range compared to those reported for the classic L-YES (ED_50_ = 170 ng [16] and 90 ng [66] using the McDonnell strain [25, 26], absolute amounts represented by ED values were calculated according to EC_50_ times test volumes used in the assays). This finding has also relevance for the calculation of mixture effects. This is of importance for the model of concentration addition which is the common method for mixture toxicity calculation with respect to the activation of the estrogen receptor. The mixture modelling according to concentration addition requires complete and thus unaffected dose–response relationships [21–23]. In case of micro-well based assays, a pragmatic way to deal with incomplete concentration response curves is to use the maximal effect level of a reference compound to define the 100% effect. However, this assumes that the maximum effect level of the reference compound equals that of the compound of interest. This might be true for a number of compounds but could lead to erroneous calculations in case of, e.g. partial receptor agonists. The possibility to differentiate partial agonists and a cytotoxic affection is an advantage of the planar in vitro assays compared to the assays based on microwell plates that can be exploited by the approach proposed in this work.

Our method was also applied to test the estrogenicity of an elastomer building material leachate (Fig. 6). As observed for BPA, the data based on the modelled signals showed smaller confidence intervals compared to the measured data with the onset of cytotoxicity. Thus, using the modelled signals rather than the measured signals results in a higher precision. For this example, no complete dose–response relationship was established; however, differences in the effect quantification are evident proving the applicability of the proposed method to extrapolate cytotoxically affected signals.

The deviations between unmodelled and modelled, unaffected peaks in both, the BPA and the elastomer leachate experiments did not exceed the maximum mean deviation of 3%. In addition to the successful validation (see “Validation” section), this emphasises that the present approach of the signal modelling produces comprehensible results. The unmodelled data normally do not provide enough basis to permissibly calculate effect values. Note that the presentation of these results in Fig. 4 was intended to illustrate the problem of information loss through cytotoxic masking. This also applies to the real sample experiment (see “Extract of the eluted elastomer” section, Fig. 5).

### Possible applications of the approach

Assessing the risk of complex mixtures which occur frequently in environmental samples, such as those leached from building materials [66–70], is a challenge due to the problem of cytotoxic masking. Planar in vitro assays may provide a tool to test such samples, as these types of bioassays are able to simultaneously identify specific effects and cytotoxic sample fractions. Additionally, extrapolation of cytotoxicity affected signals could be useful in case of effect screening or single replicate testing to avoid wasting extract volumes. As a further development, modelling of the effects could also be used to assess cytotoxicity and specific effects simultaneously, as Frische et al. [10] showed for the L-YES assay. In the case of planar assays, the affected signal peak could be used to quantify the cytotoxicity alongside the modelled signal peak, by quantifying the integral of the cytotoxic area within the modelled peak.

Besides the evaluation of signals in planar in vitro assays affected by cytotoxicity, there might be other fields of application for split peaks, peak plateaus (cut-off peaks) or other effects like sensor saturation in chemical analysis [71–74]. In the case of plateaus, the remaining data may provide enough information through their outer legs to extrapolate disturbed peaks. In the case of split peaks, the underlying reasons must be considered, as the extrapolation should only be performed on signals that were affected in height or cut off. A similar instance of sensor saturation with resulting peak plateaus occurred for motion tracking with inertial measurement units (electronic components for the measurement and detection of moving objects) [73]. The authors state two different ways of extrapolating plateaus: first, they mention triangle approximation and second quadratic approximation. Both ways are alternatives to our proposed approach. In future, further peak functions as the Chesler-Cram [42, 75, 76], the Bi-Gaussian [42, 43, 77] or the Fraser-Suzuki [42, 78] function could be implemented and validated by using the approach developed in the present study. These could be used for other planar in vitro assays and all signals with fronting or tailing. For this, however, the respective functions have to be validated for the corresponding method. However, the proposed approach could be limited by insufficiently separated signals. In this case, the choice of the right mobile phase is of crucial importance [79]. In addition, it should also be considered to use signals that clearly can be separated from the background.

## Conclusions

The masking of specific effects in in vitro assays by cytotoxicity is a commonly known phenomenon. Until now, there has been no approach to quantify cytotoxically affected signals in planar in vitro assays. The present study provides a validated approach to overcome this problem and extrapolate the actual effects of cytotoxically affected signals. By this means, a prediction of the actual, specific response was possible and resulted in a more robust evaluation of the dose–response relationship as demonstrated for the activation of the estrogen receptor. It was possible to calculate reliable dose–response relationships and corresponding ED_x_ values using the Lorentzian function. The integrals of fitted peaks did not exceed the deviation limits, with a maximum difference of only 8% between measured ideal signals and modelled affected ring signals. The approach offers a wide range of further possible uses including the parallel quantification of cytotoxicity and specific effects and could also be used to extrapolate affected peak signals resulting from other analytical methods, such as sensor saturation in chemical analysis. The importance of being able to quantify correct toxicity signals is emphasized when calculating relative potency and mixture toxicity, as whole dose–response curves are required for this purpose. Additionally, extrapolation could be useful in case of effect screening or single replicate testing to avoid wasting extract volumes. The evaluation software for the underlying methodology is freely available under the GitHub link, https://github.com/bafg-bund/extrapolation-cytotoxic-masking, and can also be used solely for the creation and integration of chromatograms from images.

## Supplementary Information

Below is the link to the electronic supplementary material.

Supplementary file1 (PDF 440 K-B)
