# Supplementary material for: Extrapolation of cytotoxic masked effects in planar in vitro assays
Source: Anal Bioanal Chem. 2024 Apr 24;416(15):3519–32. doi: 10.1007/s00216-024-05302-z (PMC11525312; doi:10.1007/s00216-024-05302-z)
Supplement: Supplementary file 1 — Supplementary file1 (PDF 440 K-B) [file 216_2024_5302_MOESM1_ESM.pdf]

Supplementary information

Extrapolation of cytotoxic masked effects in planar *in vitro* assays

Analytical and Bioanalytical Chemistry

Timothy Rosenberger[a], Anna Maria Bell[a], Georg Reifferscheid[a], Kilian Eric Christopher Smith[b], Andreas Schäffer[c], Thomas Ternes[a], Sebastian Buchinger[a]

[a] Department G - Qualitative Hydrology, Federal Institute of Hydrology (BfG), Am Mainzer Tor 1, D-56068 Koblenz, Germany

[b] Environmental Chemistry - Department of Water, Environment, Construction and Safety, University of Applied Sciences Magdeburg-Stendal, Breitscheidstraße 2, D-39114 Magdeburg, Germany

[c] Institute for Environmental Research, RWTH Aachen University, Kackertstraße 10, D-52072 Aachen, Germany

Contact: Sebastian Buchinger, Federal Institute of Hydrology (BfG), Am Mainzer Tor 1, D-56068 Koblenz, Germany, buchinger@bafg.de

List of Figures

Fig S1 *(a) Image of 10 effect signals of BPA from a p-YES with extended incubation time from 3 to 20 h. Concentrations ranging from 10 ng to 5 µg. No chromatographic development was performed. The image shows the signal detection with fluorescence imaging at $\lambda_{excitation}$ = 366 nm. (b) Corresponding image of the subsequent resazurin assay signals according to Riegraf et al. [18]. The image shows the signal detection with simultaneous incident and transmitted light* 3

Fig S2 *P-values of Chi squared tests from the fits of the ideal peaks of different functions. Whiskers show the last value within the limit of the corresponding quartile expanded by the 1.5-fold interquartile range. Boxes show the first and third quartiles, dashes show medians, dotted dashes show means and hexagons indicate values outside the 1.5-fold inter quartile range. n = 42 for Gaussian, Lorentzian and mod Gaussian, n = 8 for log-normal* 4

Fig S3 *Ratios of fit- and measured integrals by varying the percentage proportion of the outer legs of affected peak signals. The closer the ratio is to the value of 1.0, the better the alignment of the fitted peak function integral to the measured integral. The maximum range of the data for the peak fitting was defined from the nearest maximum of the inflection point to the nearest minimum – this range was set at 100 %. From the positions of inflection point, fits were then calculated on the percentage of the maximum range in steps of ten. Boxes show the first and third quartiles, whiskers show the interquartile range, dashes show medians and dotted dashes show means. The optimal ratio of fit- to ideal signal integrals of 1.0 is marked with a green dashed line. n = 42 for each value* 5

List of Tables

Table S1 *Concentrations of the used estrogen mixture and bisphenol A solutions* 2

Table S2 *Boxplot values corresponding to results of the chi squared test performed for all 42 optimal peaks and each of the four peak functions* 4

Table S3 *Boxplot values corresponding to* ***Fig S3****. IQR means inter quartile range* 5

Table S4 *Boxplot values corresponding to* ***Fig 3****. a) data of ratios from peak fit- and unfitted integrals of ideal signals and b) data of ratios from fit-integrals of mathematically constructed, affected peaks and unfitted integrals of ideal signals. IQR means inter quartile range* 6

Table S5 *Underlying values of the dose-response relationship in* ***Fig 4****. Effect values resulted from effect peak integrals. Values of the modelled data, unmodelled data in relation to the modelled data (as shown in* ***Fig 4****) and unmodelled data without relation to modelled data are. Last formed the basis for calculating the effect value of unmodelled data.* 6

Table S6 *Effect value data corresponding to the dose response relationship in* ***Fig 4*** *in the section "Cytotoxicity affected signals from bisphenol A"* 7

Table S7 *Integral data corresponding to the scatter plot in* ***Fig 6*** *in the section "Extract of the eluted elastomer"* 7

Table S1 *Concentrations of the used estrogen mixture and bisphenol A solutions*

solution	1	2	3	4	5	6	7	8	9	10
unit	ng/ml	ng/ml	ng/ml	ng/ml	ng/ml	ng/ml	ng/ml	ng/ml	ng/ml	ng/ml
E1	0.2	0.4	1	2	4	10	20	24	30	40
EE2	0.02	0.04	0.1	0.2	0.4	1	2	2.4	3	4
E2	0.02	0.04	0.1	0.2	0.4	1	2	2.4	3	4
E3	2	4	10	20	40	100	200	240	300	400
solution	1	2	3	4	5	6	7	8	9	10
unit	µg/ml	µg/ml	µg/ml	µg/ml	µg/ml	µg/ml	µg/ml	µg/ml	µg/ml	µg/ml
BPA	2.0	3.9	7.8	15.6	31.3	62.5	125	250	500	1000

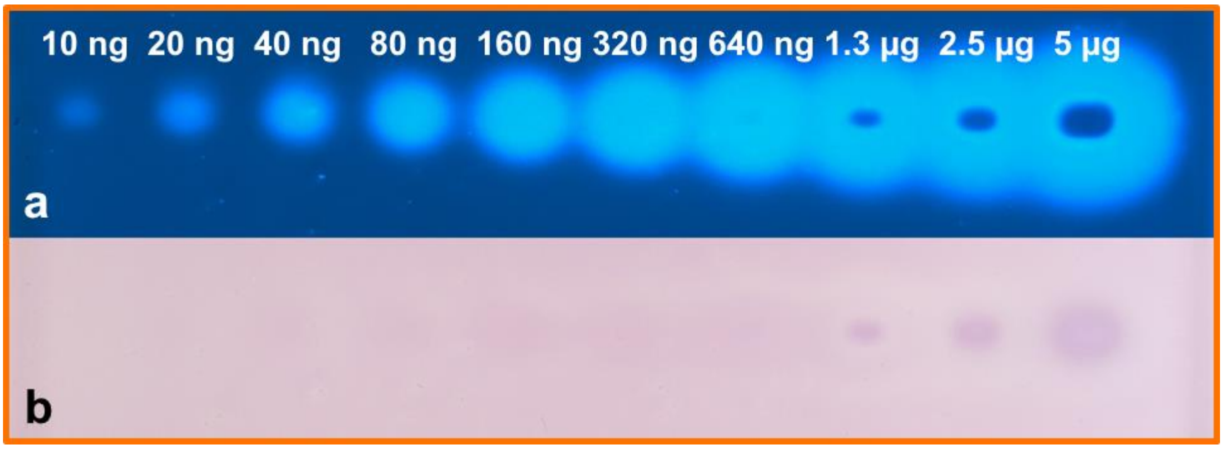

Fig S1 *(a) Image of 10 effect signals of BPA from a p-YES with extended incubation time from 3 to 20 h. Concentrations ranging from 10 ng to 5 µg. No chromatographic development was performed. The image shows the signal detection with fluorescence imaging at* $\lambda_{excitation}$ *= 366 nm. (b) Corresponding image of the subsequent resazurin assay signals according to Riegraf et al. [18]. The image shows the signal detection with simultaneous incident and transmitted light*

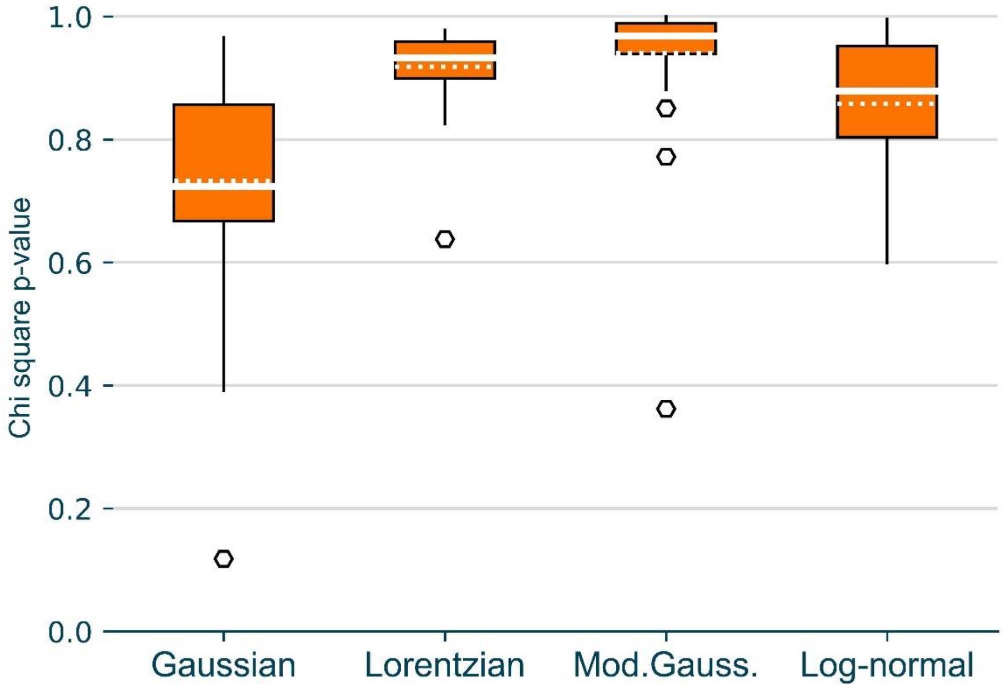

Fig S2 *P-values of Chi squared tests from the fits of the ideal peaks of different functions. Whiskers show the last value within the limit of the corresponding quartile expanded by the 1.5-fold interquartile range. Boxes show the first and third quartiles, dashes show medians, dotted dashes show means and hexagons indicate values outside the 1.5-fold inter quartile range. n = 42 for Gaussian, Lorentzian and mod Gaussian, n = 8 for log-normal*

Table S2 *Boxplot values corresponding to results of the chi squared test performed for all 42 optimal peaks and each of the four peak functions*

function	lower whisker	1st quartile	median	mean	3rd quartile	upper whisker	inter quartile range
Gaussain	0.3918	0.6673	0.7234	0.7332	0.8566	0.9656	0.1893
Lorentzian	0.8253	0.8995	0.9328	0.9187	0.9593	0.9780	0.0598
mod.Gaussian	0.8809	0.9390	0.9682	0.9410	0.9890	0.9996	0.0500
log-normal	0.5987	0.8034	0.8792	0.8584	0.9516	0.9957	0.1483

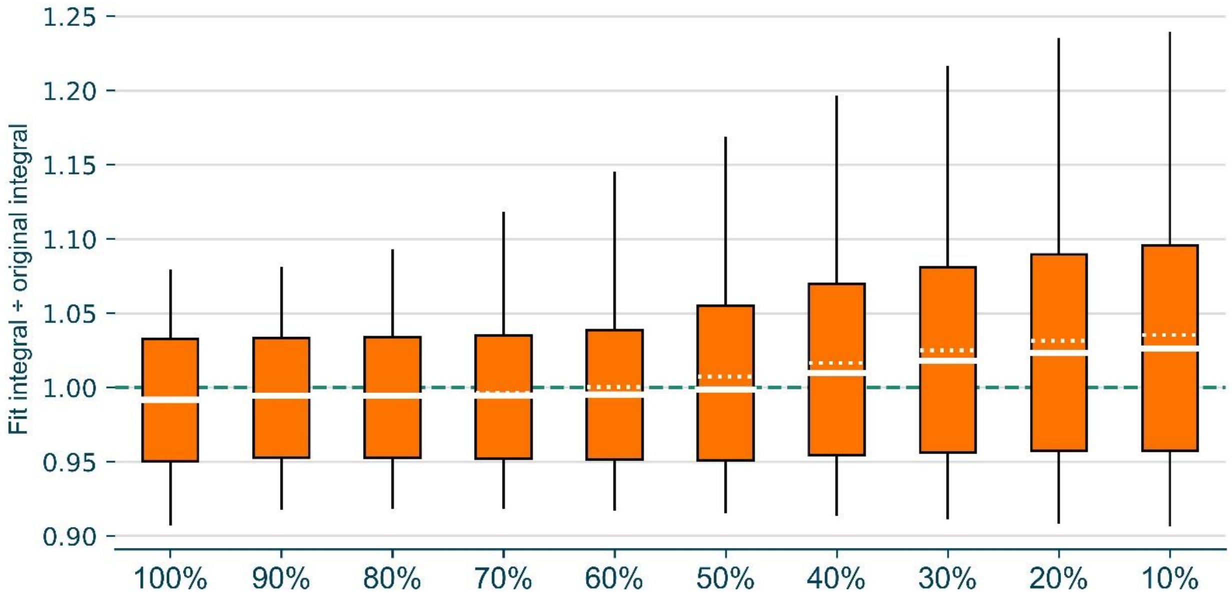

Fig S3 *Ratios of fit- and measured integrals by varying the percentage proportion of the outer legs of affected peak signals. The closer the ratio is to the value of 1.0, the better the alignment of the fitted peak function integral to the measured integral. The maximum range of the data for the peak fitting was defined from the nearest maximum of the inflection point to the nearest minimum – this range was set at 100 %. From the positions of inflection point, fits were then calculated on the percentage of the maximum range in steps of ten. Boxes show the first and third quartiles, whiskers show the interquartile range, dashes show medians and dotted dashes show means. The optimal ratio of fit- to ideal signal integrals of 1.0 is marked with a green dashed line. n = 42 for each value*

Table S3 *Boxplot values corresponding to* ***Fig S3****. IQR means inter quartile range*

proportion	lower whisker	1st quartile	median	mean	3rd quartile	upper whisker	inter quartile range	variation of IQR
100%	0.9081	0.9501	0.9918	0.9910	1.0327	1.0787	0.0827	8%
90%	0.9186	0.9528	0.9946	0.9937	1.0333	1.0806	0.0805	8%
80%	0.9190	0.9525	0.9946	0.9942	1.0337	1.0924	0.0812	8%
70%	0.9189	0.9519	0.9948	0.9965	1.0348	1.1173	0.0829	8%
60%	0.9180	0.9514	0.9954	1.0004	1.0387	1.1446	0.0873	9%
50%	0.9161	0.9509	0.9989	1.0074	1.0549	1.1679	0.1040	10%
40%	0.9144	0.9543	1.0097	1.0165	1.0697	1.1956	0.1154	11%
30%	0.9123	0.9562	1.0181	1.0251	1.0808	1.2158	0.1246	12%
20%	0.9091	0.9573	1.0231	1.0314	1.0895	1.2343	0.1322	13%
10%	0.9076	0.9574	1.0261	1.0354	1.0957	1.2387	0.1383	13%

Table S4 *Boxplot values corresponding to* ***Fig 3****. a) data of ratios from peak fit- and unfitted integrals of ideal signals and b) data of ratios from fit-integrals of mathematically constructed, affected peaks and unfitted integrals of ideal signals. IQR means inter quartile range*

	function	lower whisker	1st quartile	median	mean	3rd quartile	upper whisker	inter quartile range	variation of IQR
**a)**									
	Gaussian	1.0007	1.0082	1.0125	1.0153	1.0228	1.0361	0.0146	1%
	Lorentzian	0.9997	1.0031	1.0059	1.0085	1.0121	1.0228	0.0090	1%
	mod.Gauss.	1.0004	1.0064	1.0124	1.0149	1.0225	1.0355	0.0161	2%
	log-normal	1.0008	1.0083	1.0129	1.0135	1.0201	1.0234	0.0117	1%
**b)**	function	lower whisker	1st quartile	median	mean	3rd quartile	upper whisker	inter quartile range	variation of IQR
	Gaussian	0.8873	0.9169	0.9370	0.9405	0.9651	1.0131	0.0482	5%
	Lorentzian	0.9081	0.9501	0.9918	0.9910	1.0327	1.0787	0.0827	8%
	mod.Gauss.	0.8931	0.9175	0.9386	0.9426	0.9665	1.0163	0.0489	5%
	log-normal	0.8937	0.8944	0.9168	0.9295	0.9672	0.9820	0.0728	8%

Table S5 *Underlying values of the dose-response relationship in* ***Fig 4****. Effect values resulted from effect peak integrals. Values of the modelled data, unmodelled data in relation to the modelled data (as shown in* ***Fig 4****) and unmodelled data without relation to modelled data are. Last formed the basis for calculating the effect value of unmodelled data.*

amount of BPA in ng	effect in %		
	modelled	rel. unmodelled	unmodelled
10	17.7	18.5	33.4
20	24.3	25.2	45.5
40	30.5	31.6	57.1
80	35.6	36.8	66.5
160	41.0	42.3	76.5
320	47.1	48.6	87.8
640	53.7	55.3	100.0
1300	75.8	53.6	97.0
2500	91.5	45.7	82.6
5000	94.5	39.0	70.6

Table S6 *Effect value data corresponding to the dose response relationship in* ***Fig 4*** *in the section "Cytotoxicity affected signals from bisphenol A"*

	effect level	value in ng	confidence interval	lower prediction interval	upper prediction interval
modelled	10%	1.1	0.9	-0.1	2.2
	50%	320	63	240	390
	90%	2810	870	1760	3860
unmodelled	10%	1.6	3.7	-2.9	6.0
	50%	24	17	3.8	45
	90%	950	1820	-1250	3150

Table S7 *Integral data corresponding to the scatter plot in* ***Fig 6*** *in the section "Extract of the eluted elastomer"*

amount of sample in RCF	**integral values**			
	modelled	confidence interval	unmodelled	confidence interval
0.5	3666.2	300.2	3624.8	313.9
1	9958.1	617.1	9928.9	622.4
2	14781.4	707.5	14736.9	648.2
5	18366.5	679.8	18231.7	674.1
7.5	24906.4	1486.6	22806.8	1574.1
10	26616.7	1277.7	23484.3	1770.2
15	31144.1	1148.1	24538.6	2285.3
20	32541.8	1256.0	24642.3	1835.9
